# Simulating the impact of excise taxation for disease prevention in low-income and middle-income countries: an application to South Africa

**DOI:** 10.1136/bmjgh-2017-000568

**Published:** 2018-01-05

**Authors:** Nicholas Stacey, Amit Summan, Aviva Tugendhaft, Ramanan Laxminarayan, Karen Hofman

**Affiliations:** 1 Priority Cost Effective Lessons for Systems Strengthening, School of Public Health, Faculty of Health Sciences, University of the Witwatersrand, Johannesburg, South Africa; 2 Center for Disease Dynamics Economics and Policy, Washington, District of Columbia, USA

**Keywords:** health policy, prevention strategies, public health

## Abstract

**Introduction:**

Excise taxes are policy tools that have been applied internationally with some success to reduce consumption of products adversely impacting population health including tobacco, alcohol and increasingly junk foods and sugary beverages. As in other low-income and middle-income countries, South Africa faces a growing burden of lifestyle diseases; accordingly we simulate the impact of multiple excise tax interventions in this setting.

**Methods:**

We construct a mathematical model to simulate the health and revenue effects of increased excise taxes, which is adaptable to a variety of settings given its limited data requirements. Applying the model to South Africa, we simulate the impact of increased tax rates on tobacco and beer and of the introduction of a tax on sugar-sweetened beverages (SSB). Drawing on surveys of product usage and risk factor prevalence, the model uses a potential impact fraction to simulate the health effects of tax interventions.

**Results:**

Adopting an excise rate of 60% on tobacco would result in a gain of 858 923 life-years (95% uncertainty interval (UI) 480 188 to 1 310 329), while adopting an excise rate of 25% on beer would result in a gain of 568 063 life-years (95% UI 412 110 to 775 560) and the adoption of a 20% tax on SSBs would result in a gain of 688 719 life-years (95% UI 321 788 to 1 079 653).

**Conclusion:**

More aggressive excise tax policies on tobacco, beer and SSBs in South Africa could result in meaningful improvements in population health and raised revenue.

**Key questions**

**What is already known about this topic?**

- The literature is dominated by studies of tobacco taxation with much focus on retrospective evaluation of tobacco policy in South Africa.
- These studies find that recent tobacco taxation has seen increases in price and a fall in smoking prevalence.
- Only one study attempts to simulate the health impact of tobacco taxation, while none exist for alcohol taxation. Some more recent studies simulate the impact of sugar-sweetened beverage (SSB) taxation focusing on specific disease outcomes.

**What are the new findings?**

- This study departs from the literature by prospectively evaluating the potential impact of multiple tax interventions using a consistent modelling approach.
- This study provides the first attempt to synthesise existing evidence to simulate the impact of excise tax policies across products in South Africa and to report results using a comparable population health outcome measure.

**Recommendations for policy**

- In conjunction with the existing literature, our results suggest that while South Africa has seen some success with excise taxation, there are significant population health gains to be made with higher tax rates on tobacco and beer and with the introduction of tax on SSBs.
- Revenue raised from these interventions while small relative to aggregate public receipts could be targeted to further amplify disease treatment or prevention efforts.
- The arguments made by vested interests against these policy changes need to be evaluated relative to the potential benefits this study has identified.

## Introduction

Non-communicable chronic diseases (NCDs) are emerging as a significant cause of morbidity and mortality in South Africa.^1 2^ The country, however, faces a unique epidemiological transition trajectory, with a quadruple burden of disease that includes chronic infectious diseases, in the form of HIV/AIDS and TB, compounded by high rates of maternal and child mortality and violence and injury.

Poor recent macroeconomic performance, in particular stagnant economic growth and a relative fall in corporate income tax revenue has resulted from the global financial crisis. The consequent re-emergence of a structural deficit has placed additional constraints on the extent to which public resources can be allocated to manage these intersecting epidemics. Moreover, the structure of the South African health system mirrors its economic inequality with high-quality expensive healthcare provided to approximately 15% of the population covered by private health insurance while the remaining 85% depend on an overburdened and under-resourced public healthcare system.^1 3^

South Africa aims to deliver universal health coverage by 2025 through a National Health Insurance Scheme (NHI) but its successful implementation will rely on addressing the contextual constraints described above. Cost-effective prevention of non-communicable disease must use population-level policy tools targeting social determinants and not simply costly individual-level health service interventions.^4^ These tools include fiscal instruments such as taxes and subsidies, which can influence the price and affordability of products related to risk factors. Ideally fiscal policies would be implemented alongside complementary regulatory measures related to advertising and informative product labelling. While taxation of tobacco and alcohol has been in place to varying degrees internationally and in South Africa, there is now growing global interest in the use of fiscal tools as a means to improve diet and to reduce diet-related non-communicable disease.^5^ The WHO has called for global action to curtail the impact the of sugary drink consumption including the levying of taxes on these beverages.

South Africa’s experience with excise taxation is mixed. While the prevalence of smoking has fallen in South Africa, recent population survey estimates suggest that 18.2% of adults still identify as current smokers. Since the early 1990s, South Africa has committed to an aggressive tobacco taxation policy generally regarded as a public health success with both taxes and tobacco prices rising significantly and consumption and smoking prevalence falling.^6^ However, in recent years, tax increases have been muted with a concomitant slowing in progress on tobacco consumption reduction. With an effective excise benchmark rate of 40% of retail price of the most popular brand, South Africa ranks far below the Framework Convention on Tobacco Control and WHO’s recommendation of excise taxes constituting 70% of the price of the most popular brand of cigarettes.^7^

Despite significant alcohol intake and alcohol-related harms stretching across South Africa’s quadruple burden of disease, excise taxes on alcohol have lagged behind those on tobacco. Of consequence is the rate on beer, which disproportionately contributes to alcohol intake in the South African population. Excise rates on beer are set based on a 23% of retail price benchmark and unlike tobacco have not increased significantly, with only a ZAR 1.18 per litre real increase since 2002. The volume of beer sold has risen from 2812 to 3565 million litres in this period.^8^

In recent years (2003–2012), obesity prevalence in South Africa has increased among men by 2%, from 9% to 11%, and among women by 12%, from 27% to 39%, with a contemporaneous rise in sugar-sweetened beverage (SSB) intake.^9 10^ In response to rising obesity prevalence and diet-related disease, a tax on sugary beverages has been proposed but is yet to be legislated or implemented.^11^ Consumption of SSBs is closely linked to the onset of obesity and associated metabolic conditions.^12–14^

Collectively, the taxation of cigarettes, beer and SSBs target a significant source of the non-communicable disease burden. The Global Burden of Disease study attributes 4.96% of disability-adjusted life years (DALYs), 4.60% of DALYs and 5.98% of DALYS in South Africa to smoking, alcohol intake and high body mass-index, respectively. This study synthesises the available evidence on product use, risk factor prevalence, price responsiveness and mortality risk in a consistent mathematical modelling framework to produce estimates of the health and revenue gains of alternative excise tax policies on tobacco, alcohol and SSBs for South Africa. The findings suggest that in South Africa and other settings with similar constraints on public finances and healthcare resources, excise taxes could provide a means to simultaneously prevent disease, improve population health and raise revenue.

## Methods

### Modelling overview

Building on the health impact assessment and modelling literature prospectively estimating the impacts of population-level interventions, this study provides estimates of the impact of tobacco, alcohol and SSB tax policy scenarios on population health outcomes in South Africa over a prospective 30-year period.^15–18^ The approach incorporates economic simulation of consumption changes in response to tax-induced price changes and epidemiological simulation of the change in mortality associated with reduced use of the taxed products. A detailed mathematical description of the modelling is provided in online supplementary appendix.

10.1136/bmjgh-2017-000568.supp1 Supplementary file 1

**Figure 1:**
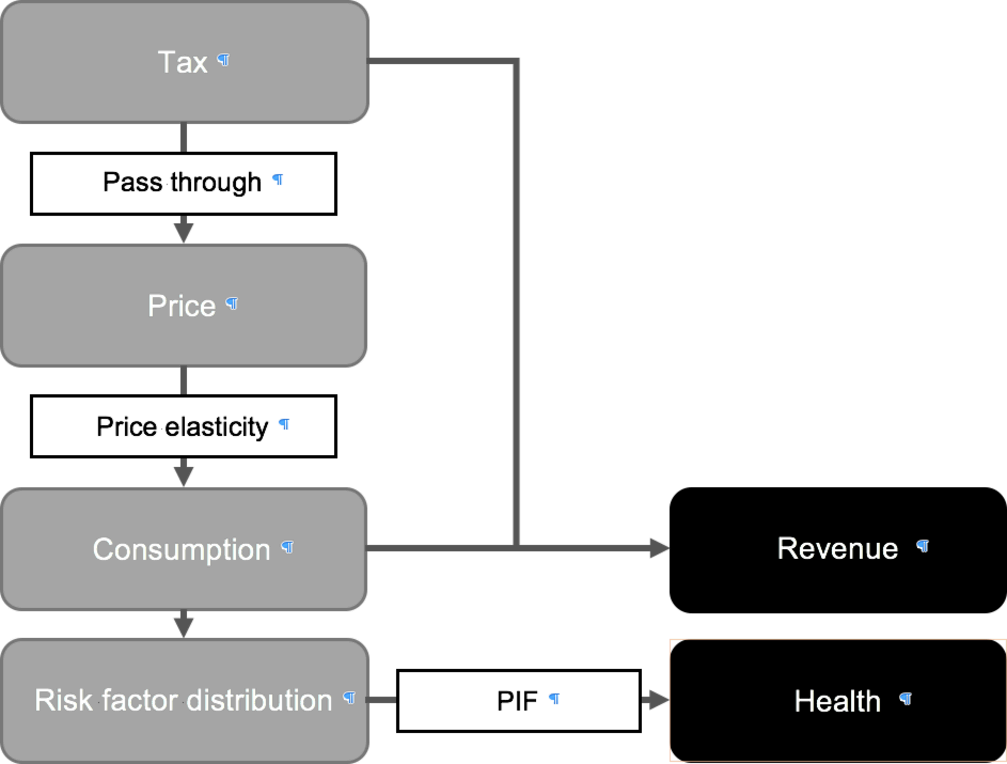
Model structure.

An overview of the model’s structure is presented in figure 1. The model begins by including a tax on the particular product and assuming the tax will be passed through to the retail price facing consumers. Existing evidence from South Africa and other low and middle-income countries (LMIC) suggests that excise taxes are entirely or indeed overshifted to consumers.^19 20^ To quantify the change in consumption resulting from the tax-induced price change, we use price elasticities. A price elasticity is a unitless measure of the proportionate change in the demand of a product for a proportionate change in its price.^21–23^ Using estimated tax-induced price changes and own-price and cross-price elasticities, we simulate reductions in consumption of the taxed product and potential substitute or compliment products (see Section 1.3 of the online supplementary appendix).

The simulated changes in consumption of the targeted products lead to shifts in the distribution of a related risk factor. Reductions in SSB consumption lead to reductions in energy intake and in body-mass index, reductions in beer intake leads to reductions in absolute alcohol intake and reductions in cigarette consumption lead to changes in the prevalence of current smoking and former smoking. For beer and SSBs, we allow for substitution to other products (ie, beer to wine or spirits and SSBs to diet beverages, water and milk). We account for gender differences in product usage through the use of gender-specific baseline distributions of consumption and risk factors.

Drawing on estimates of all-cause mortality relative risks, we construct potential impact fractions (PIF) to adjust prevailing age-specific and gender-specific mortality rates, providing estimates of mortality in the presence of the tax scenario and its induced behaviour change (see Section 1.4 of the online supplementary appendix). A baseline population projection is constructed, assuming maintenance of the status quo. A simulation population projection is constructed, employing the PIF-adjusted age-specific and gender-specific mortality rates. The differences in mortality outcomes (deaths and life-years) between the baseline and simulation projections provide an estimate of the intervention effect. We discount the life-year outcomes assuming a discount rate of 3% (we report results assuming various discount rates in Section 3 of the online supplementary appendix). We do not incorporate fertility into these projections and thus the resulting impact is conservative and should be thought of as accruing only to the current South African population. Data sources are presented in table 1. Uncertainty arising from the statistical variation in input parameter estimates is addressed by Monte-Carlo simulation. We report the mean of the resulting distribution as the point-estimate of the outcomes of interest and the 2.5th percentile and 97.5th percentiles as a 95% uncertainty interval (UI). The models were implemented in Microsoft Excel 2016 (with Visual Basic for Applications).

**Table 1:** Data sources

Model input	Source
**General**	
Population	
Age-sex structure	Statistics South Africa
Age-specific mortality rates	IHME GBD 2015
Price and tax	
Product prices	Statistics South Africa, National budget review
Existing excise duties	National budget review
**Intervention-specific**	
Cigarettes	
Price elasticity	IARC
Smoking prevalence and intensity	National income dynamics study
Mortality relative risk	Gettert *et al* (2012)
Beer	
Price elasticity	Authors’ calculations
Alcoholic beverage intake	All media and products survey
Mortality relative risk	Di Castelnouvo *et al* (2006)
SSB	
Price elasticity	Cabrera-Escobar *et al* (2013)
Beverage intake	All media and products survey
Age-sex BMI distributions	National income dynamics study
Mortality relative risk	Freedman *et al* (2006)

Details on parameter values and further mathematical detail are provided in online supplementary appendix.

BMI, body mass index; SSB, sugar-sweetened beverage.

### Scenarios modelled

For each of the taxation interventions studied, we simulate the health benefits for three different tax rates: low, medium and high. The particular interventions as well as scenario rates were identified through a series of consultative meetings convened with policy-makers and experts in the South African policy environment and with the goal of guiding study of the potential for fiscal policy interventions to improve health. The scenarios modelled are presented in table 2. For each product, the rates for the various scenarios correspond to a tax burden relative to retail price expressed in percentage terms. Such percentage of retail price benchmarks is used for setting excise rates in South Africa and elsewhere. Various products would fall under the banners of tobacco, alcohol or SSBs. As such, we focus our modelling on the particular products highlighted in table 2. While all forms of tobacco are taxed, the most common form of tobacco consumption is cigarette smoking.^24^ We focus on beer as the largest source of absolute alcohol intake among alcoholic beverages and on non-diet carbonated soft drinks as the largest source of sugar intake across soft drink types.^10^

**Table 2:** Scenarios modelled

Product	Baseline	Intervention scenario		
		Low	Medium	High
Cigarettes	40%	50%	60%	70%
Cigarettes (ZAR per 20)	12.42	19.64	32.07	58.52
Beer	23%	25%	27%	29%
Beer (ZAR per litre AA)	73.05	80.36	91.31	102.27
Sugar-sweetened beverages	0%	10%	20%	30%
Non-diet carbonated drinks (ZAR per litre)	00.00	0.78	1.56	2.34

Baseline rates taken from the national budget review.

AA, absolute  alcohol.

## Results

The interventions modelled find significant gains in years of life lived through reductions in premature mortality, as reported in table 3. For cigarettes, the adoption of the medium scenario, a 60% rate, would result in a gain of 858 923 life-years (95% UI 480 188 to 1 310 329). For beer, the adoption of the medium scenario, a 27% rate, would result in a gain of 568 063 life-years (95% UI 412 110 to 775 560). And for SSBs the medium scenario, a 20% rate, would result in a gain of 688 719 life-years over 30 years (95% UI 321 788 to 1 079 653). Across the three interventions the health effects are increasing in the rate adopted. For instance, for the SSB low scenario the gain in life-years rises from 340 408 (95% UI 144 787 to 526 862) to 953 158 (95% UI 432 661 to 1 547 834) in the high scenario. This result arises from the assumption of a constant elasticity and a greater tax inducing a greater price increase.

**Table 3:** Cumulative life-years gained across modelled interventions after 30 years

Product	Intervention scenario		
	Low	Medium	High
Cigarettes			
Male	220 998 (126 649 to 349 480)	605 807 (324 376 to 948 893)	1 393 789 (745 307 to 2 129 197)
Female	94 100 (55 813 to 135 365)	253 116 (143 604 to 373 964)	594 876 (330 802 to 871 061)
Total	315 099 (187 503 to 483 521)	858 923 (480 188 to 1 310 329)	1 988 664 (1 085 734 to 2 922 101)
Beer			
Male	139 135 (114 231 to 164 851)	343 960 (283 361 to 407 798)	543 349 (445 694 to 652 232)
Female	90 643 (39 022 to 160 670)	224 102 (93 177 to 419 431)	343 170 (144 342 to 654 324)
Total	229 778 (170 284 to 305 316)	568 063 (412 110 to 775 560)	886 520 (647 342 to 1 210 169)
SSB			
Male	168 135 (−3348 to 323 562)	353 577 (39 271 to 691 852)	460 957 (36 850 to 946 926)
Female	172 272 (97 546 to 272 522)	335 142 (178 734 to 512 763)	492 202 (254 670 to 761 928)
Total	340 408 (144 787 to 526 862)	688 719 (321 788 to 1079 653)	953 158 (432 661 to 1547 834)

Uncertainty interval in brackets. We assume a discount rate of 3%.

SSB, sugar-sweetened beverage.

The use of the targeted products is more prevalent among men than women.^15 24 25^ For the tobacco and alcohol interventions, this is reflected in the resulting health gains. For example, under the tobacco medium scenario, the gain in life-years is 605 807 (95% UI 324 376 to 948 893) among men, but only 253 116 (95% UI 143 604 to 373 964) among women. The exception to this pattern occurs with SSBs, where the gain in life years is approximately equal across males and females. This likely arises as while SSB intake is greater among men, body mass index is generally greater among women.^26^

The modelling suggests that non-trivial revenues could be raised from the interventions studied. We report estimates of changes in annual excise revenue from the taxation scenarios in table 4. In the case of cigarettes, the gains in revenue decrease from 12 897 million ZAR (95% UI 8023 to 17 761) under the medium scenario to 7 628 million ZAR (95% UI −14 548 to 28 423) under the high scenario. This arising from gains in per unit revenue being offset by falling consumption.

**Table 4:** Increases in annual revenues (Million ZAR)

Product	Intervention scenario		
	Low	Medium	High
Cigarettes	6278 (5162 to 7 461)	12 897 (8023 to 17 761)	7628 (−14 548 to 28 423)
Beer	11 367 (11 356 to 11 378)	12 916 (12 888 to 12 947)	14 315 (14 266 to 14 363)
SSB	4110 (3814 to 4 413)	6513 (5736 to 7327)	7098 (5398 to 8474)

SSB, sugar-sweetened beverage.

## Discussion

The public discourse in South Africa on excise taxation is generally limited to the potential costs and political barriers to adopting such policies as is the case in other global settings. This paper provides evidence on the potential benefits of adopting various excise tax interventions against which the costs of these policies should be evaluated. The modelled shifts in behaviour induced by the tax interventions studied would avert deaths and produce gains in life-years in a meaningful fashion and at the same time raise revenues. These shifts would have a significant impact on South Africa’s growing NCD burden and would alleviate some of the strain on the healthcare system while simultaneously directing resources to other health needs.

The strengths of this study lie in the adoption of a simple but demonstrative modelling framework that is populated with local data on product usage, price responsiveness and risk factor prevalence. The results provide the first modelling evidence of alcohol taxation interventions in South Africa and contribute to a growing literature on tobacco and SSB taxation in South Africa. The framework allows the estimation of health impacts of alternative tax-product interventions through a consistent mathematical structure and is transferable to other LMIC settings through its limited data requirements. The model only requires data on product usage and prevailing mortality rates which may be aggregated or disaggregated by age and gender based on availability. Product usage is available in many LMIC settings through Demographic and Health Surveys, dietary and tobacco use surveys. Mortality data are available through the Global Burden of Disease Study. As consumption of harmful products in the high-income countries slows, corporations are shifting their focus to the low-income and middle-income countries. There is thus a significant need for analytical methods suitable for those settings that inform the adoption of appropriate disease prevention policies.

The analytical approach is subject to some weaknesses. Due to a lack of availability of disease prevalence and incidence for some diseases linked to the taxed products, we focus solely on mortality outcomes rather than a combination of morbidity and mortality. We thus underestimate the health benefit of the interventions considered and this could be meaningful considering that the illnesses averted are chronic and are potentially characterised by years lived in states of disability. Further, while we consider interventions on three separate products, we do not jointly model the impact of combinations of taxes on different products and therefore the resulting effects should be interpreted separately.

Comparisons to the international literature are not always meaningful due to differences in modelling methodology, differences in chosen outcome measure and contextual differences in setting characteristics like population size or prevailing population health. However, consistent with other studies, we find that these interventions could potentially induce significant population health gains.^27–29^

While a SSB tax remains to be implemented, existing alcohol and tobacco taxes would need to be strengthened in order to have greater impact. An often overlooked early success of postdemocracy South Africa was the adoption of an aggressive tobacco taxation policy which saw prices rise and smoking prevalence fall dramatically. In recent years, tax increases have slowed and with them slowing reductions in cigarette sales. A barrier to the pursuit of higher tobacco excise, often raised by industry, is the perceived threat of increased illicit trading of cigarettes. However, independent estimates of the size of the illicit market suggest it has remained relatively constant despite the earlier significant rises in tobacco taxes.^30^ Alcohol is linked to all of the components of South Africa’s quadruple burden of disease, with beer serving as the largest source of alcohol intake and yet is subject to only moderate tax rates and increases. By increasing both alcohol and tobacco taxes to be more aligned with WHO standards and with the introduction of a SSB tax South Africa has the opportunity to effectively intervene before the point of healthcare delivery.

While we find uniformity in the scale of the potential health gains, we do identify heterogeneities across the tax interventions that are important for advocates and policy-makers to consider. In particular, gender differentials in the incidence of the health benefits of excise taxes vary across products. Much of the existing literature has considered the equity impacts of tax interventions by purely considering differential impacts along the income or wealth distribution. However, our study suggests gendered incidence of the benefits of excise taxes is significant and bears further consideration and research.

We find that while the health impacts are uniformly increasing in the rate adopted, this is not the case for revenues. For tobacco, we find that as rates rise, initially revenues increase but as consumption continues to fall revenues begin to fall as the per unit gains in revenue are offset by reduced overall product sales. This arises from the so-called Laffer curve, where revenue raised has a hump-shaped relationship to tax rate. That health is not subject to this phenomenon is important and has differential implications for policy-makers prioritising revenue raising as compared with policy-makers prioritising population health. This effect is only observed for tobacco due to the magnitude and range of rates modelled for the different products relative to their assumed price elasticities.

The relative magnitudes of revenues raised by the interventions considered is small relative to general annual revenue raising in South Africa of over one trillion Rand. Nevertheless, the revenues raised from the individual interventions range from 5% to 10% of annual public health expenditure. This revenue could be directed to the general revenue pool or could be directed to the prevention or treatment of the diseases caused by use of the particular products. These revenue gains would be complimented by cost-savings arising from reduced healthcare utilisation.

## Conclusion

It is estimated that the proposed NHI scheme in South Africa will require expenditure of over R250 billion by 2025. The feasibility of the provision of universal health coverage under NHI in South Africa will rely in part on effective disease prevention interventions. In the broader context of South Africa’s prevailing disease environment, constrained fiscal outlook and burdened healthcare system, fiscal interventions like excise taxes provide the opportunity for policy-makers to prevent disease, raise revenue and ease the burden of public healthcare facilities while attaining the goal of universal health coverage.
