# Supplementary material for: Simulating the impact of excise taxation for disease prevention in low-income and middle-income countries: an application to South Africa
Source: BMJ Glob Health. 2018 Jan 5;3(1):e000568. doi: 10.1136/bmjgh-2017-000568 (PMC5838397; doi:10.1136/bmjgh-2017-000568)
Supplement: Supplementary file 1 [file bmjgh-2017-000568supp001.pdf]

1 Modelling Approach

1.1 Overview

To assess the potential of tax instruments for health, one needs to estimate the magnitude of the health effects of their implementation. The simulation methods we have adopted allow us to mathematically estimate the health effects associated with each of the fiscal instruments under a defined set of assumptions. The metrics used to quantify the health effects are deaths and person years lived, or life-years gained. In addition to health, the modeling framework adopted allows us to estimate annual revenue changes due to the proposed interventions.

This section outlines the approach taken to modeling the health impact of the fiscal interventions studied. The models are based on quantitative epidemiological and economic methods used in the literature to simulate population-level interventions. As discussed in the main text, the fiscal interventions discussed here are excise taxation of cigarettes, beer and sugar-sweetened beverages (SSBs). While there are some slight differences in model implementation across interventions, broadly the same modeling approach has been taken. Here we present an overview of the approach developed.

The generic structure of the models is summarized in Figure 1 below. To calculate the effect of the tax on population health and revenue outcomes, two scenarios are simulated. The first is a baseline scenario, where consumption and population conditions are as observed in the available data. The second scenario is the intervention scenario, where the tax intervention is modelled to affect consumption of the product under consideration and to have some health effect. The difference in public health and revenue outcomes between these two scenarios captures the effect of the intervention.

The construction of the intervention scenario involves first assuming a tax is levied or specified change in the level of the tax on the product under consideration occurs. The price of the product then increases by the amount the tax change is assumed to be passed through to retail prices. This change in price leads to reduced consumption of the product and a change in the prevalence of the related risk factor, which in turn alters mortality patterns in the population. This leads to a reduction in deaths and a gain in life-years.

The models are parameterized by two key statistical quantities. To quantify the effect of price changes on consumption of the targeted products, published elasticities are used. To quantify the effect of reduced consumption on mortality, potential impact fractions (PIFs) are used. The two quantities are then incorporated into life tables allowing us to simulate the effect of the interventions.

1.2 Tax and Pass-Through

A magnitude of the tax change or new tax is assumed. These are based on moving South Africa in line with international benchmarks, as in our tobacco model, or on what is feasible, as in our alcohol model, or what has been previously proposed as in our SSB model. The change this tax induces in the price of the product under consideration, product x, is moderated by an assumed multiplicative pass-through parameter as follows:

$$\Delta p_x = \Delta T_x . m$$

where Δp_x is the change in the price per unit of product x, and ΔT is the change in the tax per unit, and m is the pass-through parameter. Thus, if $m = 100\%$ the change in the price will be exactly equal to the change in the tax. We have generally assumed this to be the case. There is evidence that excise increases are over shifted to consumers, however in order to be conservative we have adopted this as our default assumption.

Figure 1 Model Structure

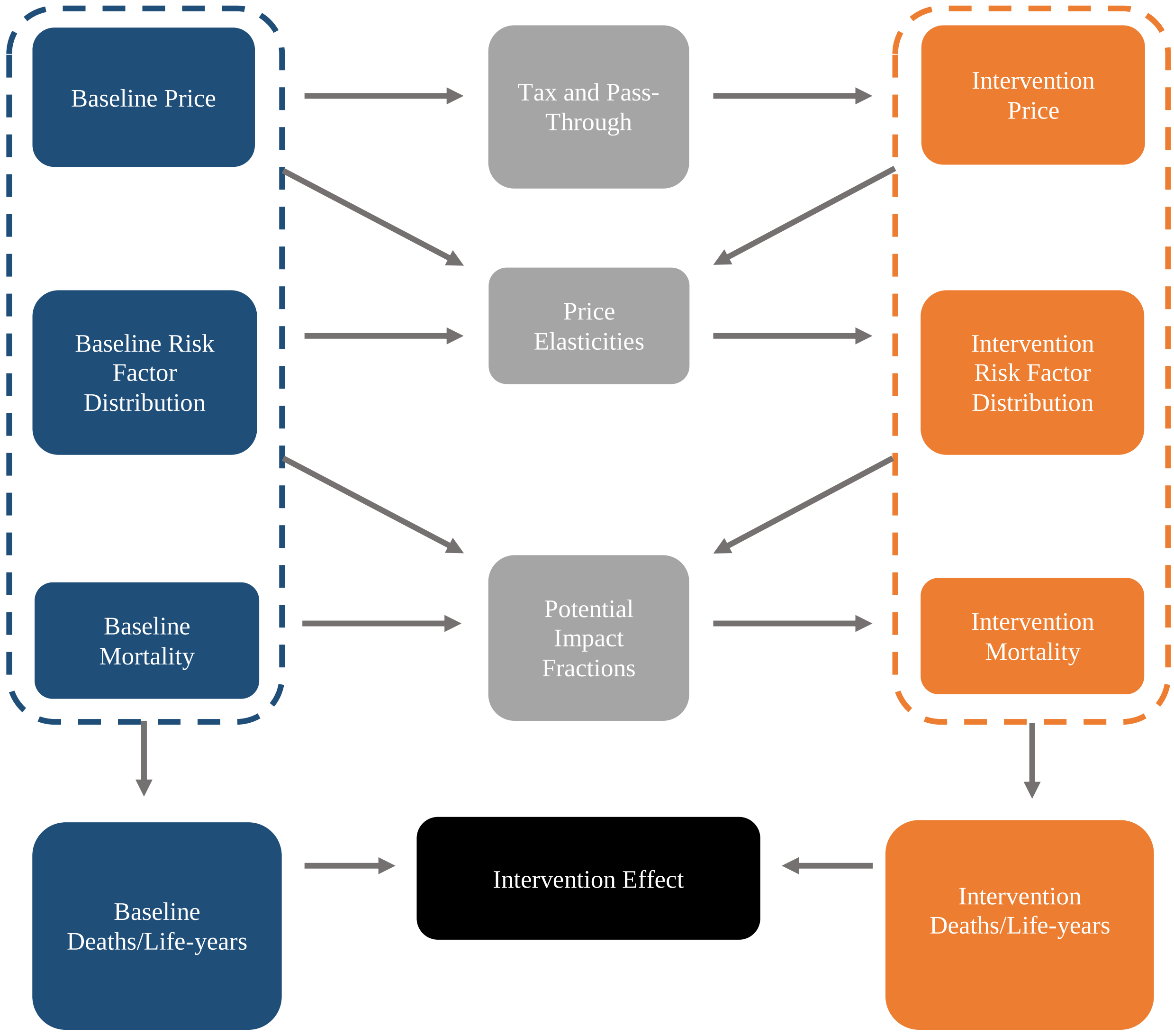

1.3 Price Elasticities

Price elasticities are statistical estimates identifying how the above identified changes in price lead to a changes in consumption of a particular product. [1, 2] An own-price elasticity measures how consumption of a good changes when its price changes, and a cross-price elasticity measures how consumption of a good changes when the price of another good changes. In this context, cross-price elasticities are important when considering tax interventions that may cause consumers of one harmful good to shift their consumption towards another. Price elasticities are defined in terms of proportionate changes. That is, they tell us by how many percent consumption of a good will reduce if the price of a good increases by a particular percent. The mathematical definition of an own-price elasticity for a product x is as follows:

$$\epsilon_{x,p_x} = \frac{\frac{\delta q_x}{\delta p_x}}{\frac{q_x}{p_x}} \approx \frac{\frac{\Delta q_x}{q_x}}{\frac{\Delta p_x}{p_x}}$$

where p_x is the price of product x and q_x is the quantity of the product x consumed. A cross-price elasticity is similarly defined but the price variable will refer to the price of a product different to the one for which the change in consumption is being considered. If reliable estimates of price elasticities are available, these can be used to infer changes in consumption for hypothetical price changes, by rearranging the above equation as follows:

$$\Delta q_x = q_x . \epsilon_{x,p_x} . \frac{\Delta p_x}{p_x}$$

This approach has been taken throughout to quantify the changes in consumption of products in response to tax-induced price changes. The change in the consumption of a harmful product the price elasticity allows us to infer, translates in a change in a particular epidemiological risk factor that affects the population health, mortality outcomes we are interested in. The risk factor is not simply the consumption of the product but the change in the consumption of the targeted product will translate to a change in the particular risk factor being modelled. For example, in tobacco model, the risk factor is simply whether or not individuals are currently smokers, former smokers or never smokes, and in the SSB model the risk factor is body-mass index.

1.4 Potential Impact Fractions

The fiscal interventions we consider here reduce consumption of the taxed products by increasing price and in so doing lead to changes in the prevalence and distribution of risk factors within the population under consideration. To quantify how the change in a risk factor's prevalence affects mortality, we use an epidemiological quantity known as the potential impact fraction (PIF). [3] The PIF allows us to estimate the percent reduction in incidence of a disease or mortality that would arise for a shift in the distribution of a particular risk factor within the population. As such PIFs are commonly used in the health-impact assessment literature to assess the health effects of population-level interventions. In our setting, we use the PIF to estimate the change in all-cause mortality. The general mathematical form of the potential impact fraction is as follows:

$$PIF = \frac{\sum_{i=1}^{n} q_i . RR_i - \sum_{i=1}^{n} p_i . RR_i}{\sum_{i=1}^{n} p_i . RR_i}$$

where q_i is the prevalence of risk factor exposure category i after the intervention of interest, p_i is the baseline prevalence of risk factor exposure category i, and RR_i is the relative risk of all cause mortality for exposure level i. In this context, the interpretation of the PIF is the percentage change in the occurrence of mortality arising due to the intervention. We use the PIFs to scale baseline age- and gender-specific mortality rates which to obtain intervention age- and gender-specific mortality rates which can be fed into life-tables to calculate our mortality outcomes of interest.

1.5 Life Tables

Once we have estimated the effect of the intervention on mortality rates, we then use discrete abridged life tables to calculate our outcomes of interest, deaths averted and years of life gained. The mortality regime of the intervention life table is calculated using the potential impact fraction discussed above. For each age-sex group the prevailing/baseline mortality rate is scaled as follows:

$$\widetilde{m}_{a,s} = \left(1 + PIF_{a,s}\right).m_{a,s}$$

where $m_{a,s}$ is the prevailing baseline mortality rate for the age a gender s population. The South African population is projected forward under these two mortality regimes, and the difference in the number of deaths and difference in years of life lived are calculated to give deaths averted and years of life gained. It should be noted that the population is projected forward without incorporating fertility, thus the results should be interpreted as only for the population that are alive today.

2 Further Detail on Each Intervention

2.1 Cigarettes

2.1.1 Consumption

Data on smoking behavior by age and gender is taken from Wave 3 of the National Income Dynamics Study (NIDS) [4]. This survey questions individuals on their current and past cigarette smoking, including asking on average how many cigarettes are smoked by current smokers. The survey does not query use of smokeless tobacco, or pipe and rolling tobacco.

2.1.2 Intervention

As cigarette smoking is the most common form of tobacco consumption in South Africa, the tax intervention is modeled only as affecting cigarettes. We assume that the price change induced by a tax change affects demand for cigarettes via the intensive (whether or not individuals smoke at all) and extensive margins (how much smokers smoke). Following the tobacco excise modeling literature [5], we assume the overall price elasticity of cigarettes is split evenly between changes in

the prevalence of current smokers and changes in the intensity of consumption amongst continuing smokers. Thus, the change in the prevalence of current smoking is given by:

$$\widetilde{CS}_{a,s} = \left(1 + \frac{1}{2}.\frac{\Delta p}{p}.\epsilon\right) CS_{a,s}$$

Note that as the change in price is positive and cigarettes own-price elasticity is negative, there will be a reduction in the prevalence of smoking. This change in the number of current smokers is modeled as due to decreased initiation and increased cessation. As such, the intervention also leads to changes in never smokers and former smokers. For age groups below 35, the intervention leads to changes in the prevalence of former smoker and never smoked as follows:

$$\widetilde{FS}_{a,s} = FS_{a,s} + \frac{1}{2}\left|\frac{1}{2}.\frac{\Delta p}{p}.\epsilon.CS_{a,s}\right|$$

$$\widetilde{NS}_{a,s} = NS_{a,s} + \frac{1}{2}\left|\frac{1}{2}.\frac{\Delta p}{p}.\epsilon.CS_{a,s}\right|$$

However, as smoking initiation is largely completed by age 35, for age groups over 35, the intervention does not affect initiation and the number of never smokers, thus the reduction in current smokers is attributed entirely to cessation. Thus, for age groups over 35 we attribute the reduced prevalence of current smoking to the prevalence of former smokers entirely.

2.1.3 Potential Impact Fraction

The exposures through which this intervention affects mortality are: never smoker, former smoker, current smoker. Thus the effect on age-sex specific mortality rates are estimated via PIF defined in a standard fashion. To be conservative, we assume the mortality impacts of smoking status only take effect for individual over the age of 50.

Cigarette Model Inputs and Parameters

Parameters	Value	Source
Price elasticities	-0.40 [-0.50, -0.30]	IARC [6] We assume a standard error of 0.10.
Tax rate	50%, 60%, 70%	Assumption
Tax pass through	100%	Assumption
Smoking-Mortality relative	Never smoker:	Gellert et al. [7]

risk	1.00 (Reference)<br>Former smoker:<br>1.34[1.28, 1.40]<br>Current smoker:<br>1.83 [1.65, 2.03]

**Other Inputs**	**Source**
Prevalence/Intensity	National Income Dynamics Study

2.2 Beer

2.2.1 Consumption

Data on consumption of alcoholic beverages is drawn from the All Media and Products Survey 2013 [8]. This survey questions respondents on servings of beverages consumed in the last seven days. The categories of alcohol considered here are beers, wine and spirits. For each beverage, individuals are characterized as abstainers, moderate drinkers and heavy drinkers. The population is then stratified across these categories for all beverages. Thus, for each age-sex group, the population is categorized into nine alcohol consumption groupings. For each category, the units of consumption assigned are the median units of reported consumption for all those the fall into the category. Average daily consumption for each grouping is calculated by dividing the survey reports by seven.

2.2.2 Intervention

The intervention is a change in the excise tax levels beer. As the intervention affects the demand for three different substitutable products, in quantifying the effect of our intervention we need to take into account own and cross-price elasticities. The change in consumption is effected at the age-sex-consumption group level. Thus for each type of beverage, c, if the level of consumption without the intervention is $q_c^{a,s,i}$, then with the tax in place, the level of consumption is given by:

$$\tilde{q}_c^{a,s,i} = \left(\sum\nolimits_b (1 + \frac{\Delta p_b}{p_b}.\epsilon_{b,c})\right).q_c^{a,s,i}$$

where a indexes age groups, s indexes gender, i indexes consumption category, b indexes the type of beverages, and $\epsilon_{b,c}$ are own (and cross-price) elasticities.

2.2.3 Potential impact Fractions

The risk factor through which this intervention affects mortality is grams of alcohol consumed per day. For each age-sex-consumption group, this is calculated for the reference (no-intervention) scenario as:

$$Alc^{a,s,i} = \sum_b q_b^{a,s,i}.Vol_b.AA_b.0.789$$

and in the intervention scenario as:

$$\widetilde{Alc}^{a,s,i} = \sum_b \tilde{q}_b^{a,s,i}.Vol_b.AA_b.0.789$$

Where Vol_b is the volume of a serving of alcohol b in ml, and AA_b is the absolute alcohol content of beverage b, and 0.789 is the density of alcohol in grams/ml. Thus, through the change in servings of each beverage consumed caused by the tax, the intervention leads to a reduction in daily grams of alcohol consumed for each age-sex-consumption group. Conventionally, a PIF is calculated by redistributing the population across fixed exposure levels. This is not possible in this case, as the exposure (grams of alcohol per day) is a composed of the effect of the intervention on multiple beverages. Thus rather than redistributing the population across fixed exposure levels, we calculate the PIF by holding the population distribution across exposure levels fixed and rather changing the levels of alcohol consumption in each consumption level, as shown below:

$$PIF^{a,s} = \frac{\sum_{i=1}^{9} \rho_i^{a,s}.RR^{a,s}(\widetilde{Alc}^{a,s,i}) - \sum_{i=1}^{9} \rho_i^{a,s}.RR^{a,s}(Alc^{a,s,i})}{\sum_{i=1}^{9} \rho_i^{a,s}.RR^{a,s}(Alc^{a,s,i})}$$

where $\rho_i^{a,s}$is the proportion of the age a gender s population in alcohol consumption group i. The relative risk function used to calculate the PIF is taken from Table 3 of Di Castenouvo et al. [9], a meta-analysis providing relative risk functions by age and gender in grams of alcohol consumed per day.

2.2.4 Estimating alcoholic beverage elasticities

While published price elasticities exist for alcoholic beverages, there are none that are available either for South Africa or are from meta-analyses that account for substitution across beverages. Given beer has close substitutes in wine and spirits, whose increased consumption could offset reductions in alcohol intake from reduced beer intake, it is extremely important to take into account cross-price elasticities. As such, we estimate our own elasticities, own-price and cross-price, for beer. We adopt the Almost Ideal Demand System (AIDS) approach of Deaton and Muellbauer. [2, 10] To do this we utilize the 2010/11 Statistics South Africa Income and

Expenditure Survey, merged with price data collected by the Statistics South Africa Consumer Price Index Unit. This approach has been adopted previously in the literature. [11] We estimate the household level, the following regression of each alcoholic beverage i's expenditure share on prices, p_{jh}, and total expenditure, m_h, as below:

$$w_{ih} = \alpha_i + \sum_{j \in \{b,s,w\}} \gamma_{ij} \ln p_{jh} + \beta_i \ln\left\{\frac{m_h}{a(p_h)}\right\} + \epsilon_{ih}$$

Based on the estimated parameters we calculate uncompensated price elasticities as discussed in Green and Alston. [12] The resulting estimates are summarized below. The elasticities of interest are beers' own-price elasticity, wine's elasticity with respect to beer price, and spirits' elasticity with respect to beer price. The resulting elasticities are broadly consistent with findings elsewhere, suggesting demand for beer is relatively inelastic, spirits and beer are complements and spirits and wine are substitutes. [13]

Beer Model Inputs and Parameters

**Parameter**	**Value**	**Source**
Price elasticities	Beer own-price elasticity: -0.824 [-0.834, -0.814]<br>Spirits cross-price elasticity: -0.225 [-0.246, -0.205]<br>Wine cross-price elasticity: 0.270 [0.240, 0.302]	Authors' calculations
Tax rate	25%, 27%, 29%	Assumption
Tax pass through	100%	Assumption
Mortality relative risk	Males:<br>β_1: -0.1445 [-0.1568, -0.13212]<br>β_2: 0.0388 [0.0359, 0.0417]<br><br>Females:<br>β_1: -0.1719 [-0.2080, -0.1358]<br>β_2: 0.0533 [0.0423, 0.0643]	Di Castelnouvo et al.[9]

**Other Inputs**	**Source**
Beverage Consumption	All Media and Products Survey

2.3 Sugar-Sweetened Beverages

2.3.1 Consumption

Data on the consumption of SSBs, milk, juice and diet sodas is taken from the All Media and Products Survey of 2013 [8]. This survey questions respondents on how many servings of each were consumed in the past seven days. Estimates of daily consumption of each category are calculated by dividing reported answers by seven, and aggregated at the age-sex level. Furthermore, these were aggregated into a measure of energy consumption by multiplying the reported consumption by an assumed serving size (ml) and multiplying by energy density (kJ/ml).

2.3.2 Intervention

The construction of the SSB model follows largely from Manyema et al wherein greater detail is included on the modelling approach. [14, 15]. The intervention is a tax levied on only SSBs. A pass through of 100% is assumed. Cross-price elasticities from Cabrera Escobar et al. [16] are used to calculate the change in consumption of the other categories of drink. The change in consumption of all the beverages is converted into a change in daily energy intake. Using energy balance equations this is converted into a change in average weight and average BMI.

For each age and sex group, the BMI distribution is fitted to a log normal distribution. The change average BMI due to the intervention is used to re-parameterize the log normal distributions, generating a new intervention BMI distribution for each age and sex population.

2.3.3 Potential Impact Fraction

Using the baseline and intervention parameterizations of the lognormal BMI distributions, baseline and intervention proportions of the population in discrete BMI categorizations are calculated. Using relative risks of all-cause mortality from Freedman et al. [17] for these categorizations, are then used to calculate PIFs used to scale down mortality for each age and sex group.

SSB Model Inputs and Parameters

Parameter	Value	Source
Price elasticities	SSB own-price elasticity: -1.299 [-1.089, -1.509]<br>Fruit juice cross-price elasticity: 0.388 [0.0095, 0.767]<br>Milk cross-price elasticity: 0.129 [-0.085, 0.342]	Cabrera-Escobar et al. [16]

	Diet drink cross-price elasticity:<br>-0.423 [-0.628, -1.219]	
Tax rate	10%, 20%, 30%	Assumption
Tax pass through	100%	Assumption
BMI-Mortality relative risk	Males:<br>BMI < 18.5:<br>0.90 [0.32, 2.55]<br>BMI 18.5 – 20.9:<br>1.13 [0.68, 1.88]<br>BMI 21 – 22.9:<br>1.00 (Reference)<br>BMI 23.0 – 24.9:<br>1.01 [0.73, 1.41]<br>BMI 25.0 – 26.9:<br>0.71 [0.49, 1.02]<br>BMI 27.0 – 29.9:<br>0.84 [0.58, 1.22]<br>BMI 30.0 – 34.9:<br>1.42 [0.94, 2.15]<br>BMI 35+:<br>2.82 [1.58, 5.04]<br><br>Females:<br>BMI < 18.5:<br>1.55 [1.10, 2.17]<br>BMI 18.5 – 20.9:<br>0.99 [0.80, 1.23]<br>BMI 21 – 22.9:<br>1.00 (Reference)<br>BMI 23.0 – 24.9:<br>1.02 [0.82, 1.26]<br>BMI 25.0 – 26.9:<br>1.30 [1.02, 1.65]<br>BMI 27.0 – 29.9:<br>1.36 [1.07, 1.74]<br>BMI 30.0 – 34.9:	Freedman et al. [17]

1.10 [0.82, 1.49]
BMI 35+:
2.14 [1.48, 3.09]

**Other Inputs**	**Source**
Beverage Consumption	All Media and Products Survey
BMI Distribution	National Income Dynamics Study

3 Discount Rate Sensitivity Analysis

3.1 Cigarettes

**Discount Rate**		**Intervention Scenario**		
		**Low**	**Medium**	**High**
**0%**	Male	377 385 [211 751, 0 574 988]	1 036 965 [0 606 728, 1 596 833]	2 474 260 [1 309 905, 3 866 781]
	Female	161 193 [97 729, 229 613]	443 323 [270 385, 0 625 477]	1 062 522 [0 602 687, 1 558 892]
	Total	538 578 [315 230, 0 799 346]	1 480 288 [0 893 463, 2 185 663]	3 536 781 [1 975 918, 5 317 943]
**3%**	Male	220 998 [126 649, 0 349 480]	605 807 [0 324 376, 0 948 893]	1 393 789 [0 745 307, 2 129 197]
	Female	94 100 [55 813, 135 365]	253 116 [143 604, 0 373 964]	594 876 [0 330 802, 0 871 061]
	Total	315 099 [187 503, 0 483 521]	858 923 [0 480 188, 1 310 329]	1 988 664 [1 085 734, 2 922 101]
**6%**	Male	134 011 [74 905, 0 199 837]	373 840 [0 199 593, 0 573 088]	854 032 [0 463 222, 1 304 345]
	Female	56 826	157 059	364 627

		[34 147, 81 119]	[90 894, 0 224 679]	[0 216 533, 0 525 150]
	Total	190 837	530 899	1 218 660
		[110 825, 0 275 536]	[0 302 902, 0 777 543]	[0 683 949, 1 770 927]

Notes: Uncertainty interval in brackets.

3.2 Beer

Discount Rate		Intervention Scenario		
		**Low**	**Medium**	**High**
**0%**	Male	240 535	604 712	948 895
		[198 296, 288 305]	[501 023, 0 716 188]	[775 729, 1 121 550]
	Female	157 614	394 397	620 225
		[64 978, 293 916]	[162 438, 0 733 155]	[252 723, 1 203 018]
	Total	398 148	999 108	1 569 120
		[294 597, 0 536 644]	[733 971, 1 369 506]	[1158 006, 2 177 450]
**3%**	Male	139 135	343 960	543 349
		[114 231, 164 851]	[283 361, 0 407 798]	[445 694, 0 652 232]
	Female	90 643	224 102	343 170
		[39 022, 160 670]	[93 177, 0 419 431]	[144 342, 0 654 324]
	Total	229 778	568 063	886 520
		[170 284, 0 305 316]	[412 110, 0 775 560]	[647 342, 1 210 169]
**6%**	Male	84 640	210 013	331 792
		[69 503, 100 716]	[174 850, 0 247 741]	[273 908, 0 395 216]
	Female	56 603	133 894	214 682
		[24 421, 102 336]	[55 260, 0 248 901]	[93 461, 0 387 292]
	Total	141 243	343 907	546 474
		[105 021, 0 190 368]	[257 611, 0 464 825]	[405 705, 0 725 788]

Notes: Uncertainty interval in brackets.

3.3 SSBs

Discount Rate		Intervention Scenario		
		**Low**	**Medium**	**High**
**0%**	Male	306 365	548 118	822 464
		[53 707, 555 551]	[-10 774, 1 135 671]	[-21 301, 1 615 527]
	Female	295 836	580 295	870 759

		[157 371, 472 790]	[301 235, 907 572]	[443 550, 1 310 557]
	Total	602 201	1 128 412	1 693 223
		[290 170, 938 714]	[472 399, 1 842 776]	[784 442, 2 640 248]
**3%**	Male	168 135	353 577	460 957
		[-3 348, 323 562]	[39 271, 691 852]	[36 850, 946 926]
	Female	172 272	335 142	492 202
		[97 546, 272 522]	[178 734, 512 763]	[254 670, 761 928]
	Total	340 408	688 719	953 158
		[144 787, 526 862]	[321 788, 1079 653]	[432 661, 1547 834]
**6%**	Male	104 615	201 000	295 109
		[5 974, 199 821]	[22 474, 395 178]	[17 229, 589 344]
	Female	104 846	207 313	307 650
		[56 816, 160 713]	[101 356, 327 438]	[170 146, 455 910]
	Total	209 461	408 313	602 759
		[93 941, 327 184]	[202 393, 655 304]	[274 755, 978 732]

Notes: Uncertainty interval in brackets.

References

1. Nhung N, Wilson N, Genç M, Blakely T. Understanding Price Elasticities to Inform Public Health Research and Intervention Studies: Key Issues. American Journal of Public Health. 2013;103(11):1954-61.
2. Deaton A, Muellbauer J. Economics and Consumer Behavior. Cambridge ; New York: Cambridge University Press; 1980 1980.
3. Group CSM. Modelling in Healthcare. Providence: American Mathematical Society; 2010.
4. SALDRU. National Income Dynamics Study 2012, Wave 3. In: Unit SALaDR, editor. 1.3 ed. Cape Town: DataFirst; 2015.
5. Verguet S, Gauvreau CL, Mishra S, MacLennan M, Murphy SM, Brouwer ED, et al. The consequences of tobacco tax on household health and finances in rich and poor smokers in China: an extended cost-effectiveness analysis. The Lancet Global Health. 2015;3(4):e206-e16.
6. IARC. Effectiveness of Tax and Price Policies for Tobacco Control. Lyon: International Agency for Research on Cancer, 2011.
7. Gellert C, Schottker B, Brenner H. Smoking and all-cause mortality in older people: systematic review and meta-analysis. Archives of internal medicine. 2012;172(11):837-44.
8. SAARF. All media and products survey 2013. Johannesburg: DataFirst; 2014.
9. Di Castelnuovo A, Costanzo S, Bagnardi V, Donati MB, Iacoviello L, de Gaetano G. Alcohol dosing and total mortality in men and women: an updated meta-analysis of 34 prospective studies. Archives of internal medicine. 2006;166(22):2437-45.
10. Deaton A, Muellbauer J. An Almost Ideal Demand System. The American Economic Review. 1980;70(3):312-26.
11. Stacey N, Tugendhaft A, Hofman K. Sugary beverage taxation in South Africa: Household expenditure, demand system elasticities, and policy implications. Preventive Medicine. 2017.
12. Green R, Alston JM. Elasticities in AIDS Models. American Journal of Agricultural Economics. 1990;72(2):442-5.
13. Nelson JP. Meta-analysis of alcohol price and income elasticities – with corrections for publication bias. Health Economics Review. 2013;3:17-.
14. Manyema M, Veerman LJ, Chola L, Tugendhaft A, Sartorius B, Labadarios D, et al. The Potential Impact of a 20% Tax on Sugar-Sweetened Beverages on Obesity in South African Adults: A Mathematical Model. Plos One. 2014;9(8).
15. Manyema M, Veerman JL, Chola L, Tugendhaft A, Labadarios D, Hofman K. Decreasing the Burden of Type 2 Diabetes in South Africa: The Impact of Taxing Sugar-Sweetened Beverages. Plos One. 2015;10(11):e0143050.
16. Cabrera Escobar MA, Veerman JL, Tollman SM, Bertram MY, Hofman KJ. Evidence that a tax on sugar sweetened beverages reduces the obesity rate: a meta-analysis. Bmc Public Health. 2013;13:1072.

17. Freedman DM, Ron E, Ballard-Barbash R, Doody MM, Linet MS. Body mass index and all-cause mortality in a nationwide US cohort. International journal of obesity (2005). 2006;30(5):822-9.
